# Nano‐Rosmarinic Acid Protects Against Chlorfenapyr‐Induced Testicular Toxicity Through Modulation of NRF2/HO‐1 and NF‐κB/NLRP3 Signaling Pathways

**DOI:** 10.1002/jbt.71037

**Published:** 2026-07-21

**Authors:** Ahmad Najem Alshammari, Ayat B. Al‐Ghafari, Huda A. Al Doghaither, Ahmed M. S. Hegazy, Ekramy M. Elmorsy, Asmaa Fady Sharif, Zahraa Khalifa Sobh

**Affiliations:** ^1^ Department of Medical Laboratory Technology, College of Applied Medical Sciences Northern Border University Arar Saudi Arabia; ^2^ Department of Biochemistry, Faculty of Science King Abdulaziz University Jeddah Saudi Arabia; ^3^ Experimental Biochemistry Unit, King Fahd Medical Research Center King Abdulaziz University Jeddah Saudi Arabia; ^4^ Editome: Precision Gene Editing Unit, King Fahd Medical Research Center King Abdulaziz University Jeddah Saudi Arabia; ^5^ Department of Anatomy, Faculty of Medicine Northern Border University Arar Saudi Arabia; ^6^ Center for Health Research Northern Border University Arar Saudi Arabia; ^7^ Department of Clinical Medical Sciences, College of Medicine Dar Al‐Uloom University Riyadh Saudi Arabia; ^8^ Department of Forensic Medicine and Clinical Toxicology, Faculty of Medicine Tanta University Tanta Egypt; ^9^ Department of Forensic Medicine and Clinical Toxicology, Faculty of Medicine Alexandria University Alexandria Egypt

**Keywords:** chitosan nanoparticles, chlorfenapyr, male reproductive toxicity, oxidative stress, Rosmarinic acid

## Abstract

Chlorfenapyr (CFP) is a widely used pesticide associated with significant toxicological effects, including reproductive toxicity. Rosmarinic acid (RA) possesses potent antioxidant and anti‐inflammatory properties; however, its therapeutic application may be limited by poor physicochemical characteristics. This study investigated the molecular mechanisms underlying CFP‐induced testicular toxicity and evaluated whether nano‐encapsulation of RA within Chitosan nanoparticles (RA‐CHNPs) improves its protective efficacy against CFP‐induced reproductive damage. A total of 60 adult male Wistar rats were allocated into six groups (*n* = 10/group): control, RA, RA‐CHNPs, CFP, CFP + RA, and CFP + RA‐CHNPs. Animals received oral CFP (180 mg/kg/day) and/or RA (75 mg/kg/day, crude or nano‐encapsulated) for 30 consecutive days. CFP exposure significantly reduced testicular weight, sperm count, sperm motility, luteinizing hormone, follicle‐stimulating hormone, and testosterone levels. These alterations were accompanied by oxidative stress, increased pro‐inflammatory cytokine production, apoptosis, and activation of NF‐κB/NLRP3 signaling. Immunohistochemical analysis revealed marked NF‐κB expression in spermatogenic and Leydig cells, together with strong NLRP3 expression within seminiferous tubules. Co‐administration of RA‐CHNPs was associated with a marked attenuation of these adverse effects, denoting greater protective efficacy than crude RA. RA‐CHNPs reduced NF‐κB and NLRP3 expression, which was accompanied by improved testicular architecture, restored spermatogenic activity, and normalized reproductive hormone levels. These protective effects were associated with activation of the NRF2/HO‐1 antioxidant pathway, suppression of inflammatory responses, and regulation of apoptosis‐related genes. Overall, RA‐loaded Chitosan nanoparticles provided superior protection against CFP‐induced testicular toxicity compared with crude RA, highlighting the potential of nanoformulation strategies to enhance RA's biological efficacy.

## Graphical abstract

Chlorfenapyr reduces testicular weight, sperm quality, and reproductive hormones. This study highlights that RA within chitosan nanoparticles (RA‐CHNPs) are superior to Rosmarinic acid in mitigating CFP‐induced testicular damage. RA within chitosan nanoparticles leads to the reduction of NF‐κB and NLRP3 expression in seminiferous tubules and activates NRF2/HO‐1 antioxidant pathway and controls apoptotic gene expression.

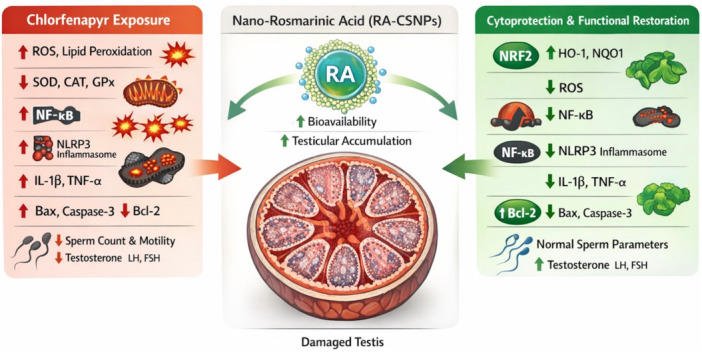

## 1 Introduction

Pesticides contribute significantly to crop productivity in contemporary agriculture by controlling insect pests. Nevertheless, the widespread unauthorized use induced deleterious ecological and health problems, particularly in human beings [1, 2]. Chlorfenapyr (CFP), an insecticide derived from pyrrole, is well‐known for its strong efficiency against several resistant pests [2]. Human exposure to CFP primarily occurs through oral ingestion, including contaminated food and water [3].

Accumulating evidence indicates that CFP can adversely affect male reproductive function. Experimental studies have demonstrated reductions in sperm production, impaired testosterone synthesis, and structural damage to testicular tissue following CFP exposure [4, 5]. Exposure to CFP has been associated with increased oxidative stress and alterations in metabolic parameters [6, 7, 8]. Excessive generation of reactive oxygen species (ROS) could disrupt spermatogenesis and steroidogenesis [9, 10]. Therefore, oxidative stress was suggested as a key mechanism underlying CFP‐induced testicular injury [11]. However, the molecular pathways linking CFP exposure to testicular dysfunction remain incompletely understood.

Natural antioxidants are being studied for their ability to combat oxidative stress and cellular impairment triggered through exposure to xenobiotics in various tissue including testicles [12, 13, 14, 15, 16]. Rosmarinic acid (RA) is a polyphenolic natural substance that exists broadly in Lamiaceae plants. Rosmarinic acid has potent antioxidant properties, with potential anti‐inflammatory effects. It also demonstrates anti‐apoptotic, anti‐microbial, and immune‐regulating activities [17]. In addition, RA has demonstrated protective effects in experimental models, alleviating oxidative stress in multiple body systems and organs, involving the kidneys, liver, and nervous system [18]. Furthermore, RA has been reported to improve testosterone levels and reproductive function in experimental models, supporting its potential utility in preserving male reproductive health [13, 19, 20].

Despite its therapeutic potential, RA faces challenges in clinical and experimental applications due to poor bioavailability, chemical instability, and low water solubility, which reduce its efficacy [21]. To mitigate the reported limitations, nanotechnology‐based drug delivery techniques have been developed as promising solutions. Nanocarriers provide several benefits, such as enhancing drug stability by protecting it from enzymatic and chemical breakdown, improving bioavailability, and enabling targeted delivery to specific tissues. Therefore, nanocarriers could enhance therapeutic outcomes [22, 23, 24].

A natural cationic polysaccharide called Chitosan is a promising nanocarrier due to its biodegradability, biocompatibility, non‐toxicity, low immunogenicity, and wide pharmacological applications [25, 26]. Chitosan‐based nanoparticles can effectively deliver poorly soluble compounds such as RA, promoting cellular uptake, prolonging release, and maintaining biological effects over time [21, 27]. Notably, nanoformulations administered orally are suggested to further enhance systemic bioavailability and facilitate targeted distribution to reproductive organs, thereby bridging the gap between the exposure route and organ‐specific protection [28].

To date, there is limited evidence concerning the preventive efficacy of RA‐loaded Chitosan nanoparticles (RA‐CHNPs) against CFP‐induced reproductive harm. The molecular processes underlying CFP‐induced testicular injury and the potential modulatory effects of RA‐CHNPs remain poorly defined. This work aimed to clarify the molecular mechanisms behind CFP‐induced testicular damage in male rats and to evaluate the protective effects of RA and RA‐loaded Chitosan nanoparticles. Emphasis was placed on assessing reproductive function, oxidative stress, inflammation, apoptosis, and the involvement of NRF2/HO‐1 and NF‐κB/NLRP3 signaling pathways. This study aimed to assess whether nanoencapsulating RA with Chitosan could improve its biological efficacy in mitigating CFP‐induced reproductive deficits compared with crude RA.

## 2 Materials and Methods

### 2.1 Preparation of RA‐CHNPs

Rosmarinic acid was bought from AB Chem Company, Egypt. Chitosan (Sigma–Aldrich, 190–310 kDa and 85% degree of deacetylation) was used for nanoparticle preparation. The mentioned molecular features are critical in influencing both the encapsulation efficiency of RA and the nanoparticles' physical stability. Chitosan nanoparticles were prepared by dissolving Chitosan (2 mg/mL) in a 1% acetic acid solution containing 1% Tween 80 as a stabilizing agent. The solution was sonicated for 15 min to allow homogenous dispersion and promote nanoparticle formation. Subsequently, we have adjusted the pH of the Chitosan solution to 5 using 2 N NaOH to facilitate effective nanoparticle formation.

Rosmarinic acid was incorporated at concentrations ranging from 0 to 3000 µg/mL, followed by 5 min of sonication to achieve uniform dispersion. A freshly prepared sodium tripolyphosphate (TPP) solution in deionized water was slowly stirred continuously with the Chitosan‐RA mixture. To achieve optimal crosslinking and nanoparticle formation, the Chitosan‐to‐TPP weight ratio was maintained at 5:1. The Chitosan‐RA mixture was then stirred for an additional hour to allow the TPP‐induced crosslinking reaction to complete, resulting in stable RA‐loaded nanoparticles. At 4°C, centrifugation at 12,000 × *g* for 20 min was performed to allow collecting the nanoparticles. To eliminate any unincorporated RA and residual TPP, the resulting pellet was subjected to two successive washes with deionized water. Finally, the nanoparticles were preserved by freezing at −80°C followed by freeze‐drying [29, 30].

Zetasizer NanoZS was utilized to determine the physicochemical characteristics of nanoparticles, including surface charge, hydrodynamic size, and polydispersity index (PDI). Zeta potential was measured in triplicate, and the Z‐average, representing the mean particle size, was assessed using samples suitably diluted in distilled water [31]. We have transmission electron microscopy (TEM; JEOL 2100, Japan) to determine the morphology and surface features of the nanoparticles, at an accelerating voltage of 160 kV.

Rosmarinic acid encapsulation in nanoparticles was determined by evaluating loading efficiency (LE) and loading content (LC), which allows quantifying the proportion of RA incorporated into the Chitosan matrix. Rosmarinic acid content was quantified using a calibration curve produced from the absorbance of serially diluted RA standards at 334 nm. The amount of unencapsulated RA was subsequently measured by spectrophotometric analysis of the supernatant obtained after nanoparticle preparation. Encapsulation performance was assessed by calculating LE and LC using the following equations: LE (%) = [RA in nanoparticles/RA added] × 100; LC (%) = [RA in nanoparticles/nanoparticle weight] × 100.

### 2.2 FTIR Characterization of Nanoparticles

The chemical interactions and functional groups of RA, Chitosan, and RA‐CHNPs were examined using Fourier‐transform infrared spectroscopy (FTIR; Nicolet iS10, Thermo Fisher, USA). For analysis, samples were blended with potassium bromide (KBr) at a 1:100 ratio and pressed into thin pellets. We have recorded the resulting spectra over the wavenumber range 4000–400 cm^−1^. This allowed the identification of the characteristic peaks and provided evidence of efficacious encapsulation of RA inside the Chitosan nanoparticles [32].

### 2.3 In Vitro Release Study of RA From RA‐CHNPs

The release of RA from the nanoparticles was monitored over 48 h under conditions mimicking the physiological environment. RA‐CHNPs were dispersed in phosphate‐buffered saline (PBS, pH 7.4) and incubated in a shaking incubator at 37°C. On definite intervals at 0, 1, 2, 4, 8, 12, 24, and 48 h, samples of the release medium were gathered and instantly substituted with fresh PBS to preserve sink conditions. The quantities of RA released at each interval were measured using a spectrophotometer at 334 nm, based on the previously prepared calibration curve. The cumulative release (%) was calculated to analyze the amount of released kinetics of RA from the nanoparticles over the 48 h [33].

### 2.4 Storage Stability Study of RA‐CHNPs

The storage stability of RA‐CHNPs was evaluated by monitoring their physicochemical properties over time. Samples were stored at 4°C and analyzed at predetermined time intervals (0, 1, 2, and 3 months). At each time point, particle size, polydispersity index (PDI), and zeta potential were measured using dynamic light scattering (DLS). Changes in particle size distribution and surface charge were recorded to assess any potential aggregation or instability during storage. All measurements were performed in triplicate, and the results were expressed as mean ± SD.

### 2.5 Experimental Animals

Sixty adult male Wistar rats were used, with an average body weight of 164.22 ± 9.63 g. The animals were housed in plastic cages in a standard laboratory environment, including a room temperature of 25°C, approximately 45% humidity, and a 12‐h light/dark cycle. Rats were allowed free access to food and water, and a 15‐day acclimatization period before experimentation. All animal procedures were performed following the recommendations of the Organization for Economic Cooperation and Development's (OECD), Guideline 420 (2001) for acute oral toxicity in rodents.

### 2.6 Study Design and Procedures

Rats were randomly divided into 6 groups (*n* = 10 per group). Group I (control group) received oral corn oil. Group II received RA (75 mg/kg) through oral gavage, and Group III received RA‐CHNPs at the same dose encapsulated in Chitosan nanoparticles. These dosing regimens have been confirmed to be safe for daily administration in a previous study [19]. Group IV received CFP (180 mg/kg) suspended in corn oil, corresponding to approximately one‐third of the compound's oral LD50 (commercial CFP, Corps Top 24% SC; LD50 = 544.3 mg/kg), as determined by Weil's method [34]. This sub‐lethal dose was selected in accordance with established toxicological practices to reliably induce measurable systemic toxicity and elucidate underlying mechanistic pathways, including oxidative stress and inflammation, within a controlled experimental timeframe. Although higher than typical human environmental exposure levels, such dosing is suitable to detect biological effects and evaluate the protective efficacy of interventions [35, 36]. Groups V and VI received combined treatments of CFP with either RA or RA‐CHNPs, at the same doses as previously described.

The oral route was chosen to deliver a controlled, repeatable systemic dose and to mimic the primary human exposure pathway to CFP, which is primarily through food and accidental ingestion [36]. The assessment of indirect testicular toxicity mediated by oxidative stress, mitochondrial dysfunction, and endocrine disruption is possible by the gastrointestinal absorption and systemic distribution of the pesticides and their metabolites to peripheral organs, including the testes, made possible by oral administration. This method improves the applicability of the findings to actual human exposure situations [28]. All treatments were administered orally once daily for 30 consecutive days, with at least a 30‐min interval between CFP and the other compounds.

### 2.7 Sample Collection and Biochemical Analysis

At the end of the experiment, rats were fasted for 10 h. Then, rats were euthanized under isoflurane anesthesia followed by administration of a high‐dose isoflurane overdose. Death was confirmed by the absence of respiration and heartbeat. Animals from each group were then allocated for sample collection, with seven rats for biochemical and molecular analyses and three for histopathological examinations. A retro‐orbital venous sinus puncture was made to gather blood samples into plain tubes [37]. The samples were allowed to clot at room temperature and then centrifuged for 10 min at 4000 rpm. The prepared serum samples were stored at −20°C for later hormonal assays. Following blood collection, we have carefully dissected the testes and cleared them of surrounding fat and connective tissue. Testes were blotted dry, and the testicular weight was determined as an objective parameter of reproductive health. The testes were rinsed in chilled PBS, gently dried on filter paper, and then processed for further experiments.

For histology, we have preserved one testis from each animal in 10% neutral‐buffered formalin. The contralateral testis was processed in ice‐cold Tris–HCl buffer (50 mmol/L, pH 7.4) utilizing a mechanical homogenizer. After centrifugation at 10,000 × g for 15 min at 4°C, we separated the supernatant, which was then stored at −80°C for later evaluation of oxidative and inflammatory parameters. Commercially available ELISA kits were then utilized to measure serum biochemical parameters following the manufacturers' instructions. Serum testosterone levels were assessed using a rat testosterone ELISA kit from MyBioSource (San Diego, California, USA; Cat. No. MBS766199). Additionally, serum levels of other reproductive hormones, including the luteinizing hormone (LH) and follicle‐stimulating hormone (FSH), were determined using rat‐specific ELISA kits from Cusabio, Wuhan, China (LH: Cat. No. CSB‐E12654r; FSH: Cat. No. CSB‐E06869r). All assays have been conducted according to the manufacturer's protocols.

### 2.8 Estimation of Sperm Parameters

The epididymal sperm concentration was measured using the hemocytometer method [38, 39]. The right epididymis was cut into small pieces in one mL of isotonic NaCl and left at room temperature for about 4 h to enable sperm release into the fluid. Post‐incubation, 0.5 mL of semen was taken with a dilution pipette, and 2% eosin was added until the 10‐line mark. Eventually, 10 µL of the diluted sperm suspension was ploaded into both chambers of the Thoma hemocytometer.

Using a light microscope at 400× magnification (10 × 40), the sperm were counted, and the results were reported as million sperm per right cauda epididymis. Slides were brought to 37°C on a light microscope with a heated stage. Left cauda epididymal fluid was mixed with Tris buffer and applied to the slides. Sperm motility was assessed at 400× magnification across three fields per sample. Eosin–Nigrosin staining was used to assess abnormal sperm. On a preheated slide (37°C), a mixture of sperm suspension, Tris buffer, and stain was placed. Under a light microscope at 400× magnification, 200 sperm cells per slide were assessed, and the proportion of abnormal sperms were reported [38].

### 2.9 Oxidative and Inflammatory Profiling

To assess testicular antioxidant status, the activities of catalase (CAT), glutathione peroxidase (GPx), and superoxide dismutase (SOD) were measured, along with reduced glutathione (GSH) levels, using BioDiagnostic colorimetric assay kits (Egypt). The manufacturer's instructions were followed: CAT (Cat. No. CA 2517), GPx (Cat. No. GSH‐Px 2524), SOD (Cat. No. SOD 2521), and GSH (Cat. No. GR 2511). Catalase activity was assessed by measuring the decomposition of hydrogen peroxide (H_2_O_2_) at 510 nm. The superoxide dismutase activity was evaluated by measuring its inhibitory effects on the reduction of nitroblue tetrazolium (NBT) at 560 nm. In comparison, GPx activity was assessed based on the rate of NADPH oxidation at 340 nm.

As an indicator of lipid peroxidation, Malondialdehyde (MDA) levels were assessed by means of a thiobarbituric acid reactive substances (TBARS) assay kit from BioDiagnostic, Egypt (Cat. No. MD 2529) following the manufacturer's directions. Oxidative protein damage in testicular homogenates was determined via calculating protein carbonyl (PC) content using a MyBioSource ELISA kit (USA, Cat. No. MBS760520) as per the manufacturer's instructions. Additionally, we have assessed testicular levels of tumor necrosis factor‐alpha (TNF‐α), interleukin‐1 beta (IL‐1β), and interleukin‐6 (IL‐6) using Bio‐Med Diagnostic ELISA kits (TNF‐α: CEK1115; IL‐1β: CEK1106; IL‐6: CEK1108) according to the manufacturer's protocols.

### 2.10 Enzyme‐Linked Immunosorbent Assay

An Enzyme‐Linked Immunosorbent Assay (ELISA) was used to quantify the levels of Nrf2, HO‐1, NF‐κB, and caspase‐3 proteins in rat tissue homogenates. Assay kits were purchased from a retail outlet, and the accompanying instructions were followed during use. Protease inhibitors were incorporated into ice‐cold phosphate‐buffered saline for tissue homogenization. To obtain the supernatants, the mixture was subsequently centrifuged at 10,000 × *g* for 15 min at 4°C. The Bradford test was subsequently performed to estimate total protein (TP) concentration.

To assess Nrf2 activity, we have used the Nrf2 Transcription Factor Assay Kit (Colorimetric, Abcam, Cat. No. ab207223). We examined its binding affinity to DNA in nuclear extracts. Heme Oxygenase 1 (HO‐1) levels were quantified utilizing the Rat Heme Oxygenase 1 (HO‐1) ELISA Kit (SimpleStep ELISA®, Abcam, Cat. No. ab279414). Additionally, NF‐κB (p65) activation was assessed utilizing the NF‐κB p65 Transcription Factor Assay Kit (Colorimetric, Abcam, Cat. No. ab133112). The Elabscience Rat CASP3 (Caspase 3) ELISA Kit (Sandwich ELISA, Elabscience, Cat. No. E‐EL‐R0160) was employed to determine the levels of caspase‐3 protein. This kit is specifically designed for rat samples. A microplate reader was employed to calculate absorbance at 450 nm for both standards and samples. We adopted standard curves to evaluate concentrations, which were consequently adjusted to show the total protein content (in pg/mg protein).

### 2.11 Quantification of Gene Expression

Testicular tissue samples were processed using 1 mL of QIAzol Lysis Reagent and the Tissuelyser II to extract total RNA. Following the addition of chloroform, the samples underwent centrifuge at 12,000 × g for 15 min. The aqueous layer containing RNA was precisely gathered. Isopropanol precipitation followed, along with RNA retrieval using centrifuge at 12,000 × *g* for 10 min. The extracted RNA was later resuspended in DNase/RNase‐free water, where its concentration and purity were assessed using spectrophotometric methods. cDNA synthesis was conducted using the iScript™ cDNA Synthesis Kit following the manufacturer's guidelines. Gene expression analysis was conducted via qRT‐PCR with iTaq Universal SYBR Green Supermix, utilizing primers for markers related to oxidative stress, inflammation, and apoptosis (Macrogen, Seoul, South Korea) (Table 1). To ascertain the primer integrity, the primers were initially reconstituted in nuclease‐free water. Real‐time PCR amplification was performed through a Rotor‐Gene Q system, and variations in gene expression were noted and analyzed according to the comparative Ct (2^−ΔΔCt) method [40].

**Table 1:** List of gene‐specific primer sequences utilized for qPCR amplification of oxidative stress, inflammatory, and apoptotic markers.

Gene	Sequences (5′‐3′)	Accession no	Length (bp)
*Nrf2*	F: TTGTAGATGACCATGAGTCGC	NM_031789.2	141
	R: TGTCCTGCTGTATGCTGCTT		
*HO‐1*	F: ATGTCCCAGGATTTGTCCGA	NM_012580.2	144
	R: ATGGTACAAGGAGGCCATCA		
*NF‐κB*	F: AGTCCCGCCCCTTCTAAAAC	NM_001276711.1	106
	R: CAATGGCCTCTGTGTAGCCC		
*NLRP3*	F: F: TCCTGCAGAGCCTACAGTTG	NM_001191642.1	185
	R: GGCTTGCAGCACTGAAGAAC		
*Bax*	F: TTTCATCCAGGATCGAGCAG	NM_017059.2	154
	R: AATCATCCTCTGCAGCTCCA		
*Caspase‐3*	F: ACTGGAATGTCAGCTCGCAA	NM_012922.2	270
	R: GCAGTAGTCGCCTCTGAAGA		
*BCL‐2*	F: GACTTTGCAGAGATGTCCAG	NM_016993.2	214
	R: CAGGTACTCAGTCATCCAC		
*β‐Actin*	F: CAGCCTTCCTTCTTGGGTATG	NM_031144.3	360
	R: AGCTCAGTAACAGTCCGCCT		

Abbreviations: Bax, Bcl‐2‐associated X protein; BCL‐2, B‐cell lymphoma 2; Caspase‐3, Cysteine‐aspartic protease 3; HO‐1, Heme oxygenase‐1; NF‐κB, Nuclear factor kappa B; NLRP3, NOD‐like receptor family pyrin domain‐containing 3; Nrf2, Nuclear factor erythroid 2–related factor 2; β‐Actin, Beta actin (housekeeping gene).

### 2.12 Histopathology Analysis

Testicular samples were fixed in 10% neutral buffered formalin and gradually dehydrated using several ethanol solutions ranging from 70% to 100%, each for about an hour. After dehydration, the tissues underwent two consecutive xylene treatments, each lasting 1 h, before being embedded in paraffin wax. Sections of the paraffin‐embedded testicular tissues were sliced to a thickness of approximately 5 µm and stained with hematoxylin and eosin [41]. Histopathological assessment was conducted using a light microscope, where selected areas were captured using a high‐resolution digital imaging system. A validated semi‐quantitative scoring system, referred to as Johnsen's score [42], was employed for the histopathological assessment of testicular injury. A total of 50 to 100 seminiferous tubules were analyzed in each animal, and the mean score was used to evaluate spermatogenesis health. Histological slides were assessed blindly by two independent observers without identification of the experimental group of each slide to reduce observer bias.

### 2.13 Immunohistochemical Assay of NF‐kβ and NLRP3

Five‐micrometer sections from paraffin blocks were first dewaxed and then rehydrated using series of alcohol solutions. Antigens were retrieved utilizing a 0.05 M citrate buffer at pH 6.8. Endogenous peroxidase activity was blocked with 0.3% H_2_O_2_. We have incubated the sections with primary rat antibodies against NF‐κB p65 (Invitrogen, Cat. No. PA5‐27617, 1:100) and NLRP3 (Abcam, Cat. No. ab109314, 1:500). A goat anti‐rabbit secondary antibody was utilized to incubate the sections at room temperature using the DAKO EnVision™ system (Cat. No. K4003, horseradish peroxidase‐labeled polymer) for use in rat tissue. Brown coloration was developed with DAB, followed by counterstaining with Mayer's hematoxylin. The immunostaining intensity was quantified as a proportion of positive area in 10 high‐power fields [43].

### 2.14 Statistical Analysis

Prior to the analysis, the data were assessed for normality and homogeneity of variance using the Shapiro–Wilk test and Levene's test, respectively. Statistical analyses were performed with one‐way analysis of variance (ANOVA) using SAS software (2012; PROC ANOVA), followed by Tukey's post hoc test for multiple comparisons. Results are presented as mean ± standard error, with statistical significance set at *p* < 0.05. Graphical representations were generated using GraphPad Prism version 9.0 (GraphPad Software, USA). Moreover, principal component analysis (PCA) was carried out using the freely available SRplot ‐ Science and Research online platform (https://www.bioinformatics.com.cn/en), employing the platform's default settings.

### 2.15 Ethical Consideration

The current study was launched after obtaining approval from the Ethics Committee at the Faculty of Medicine, Alexandria University (FWA number: 00018699, IRB number: 00012098, approval serial number: 0307571). The experiments conducted, along with data descriptions and statistical analyses, adhered to the ARRIVE guidelines for reporting in vivo research.

## 3 Results

### 3.1 Physicochemical Characterization of RA‐CHNPs

**Figure 1:**
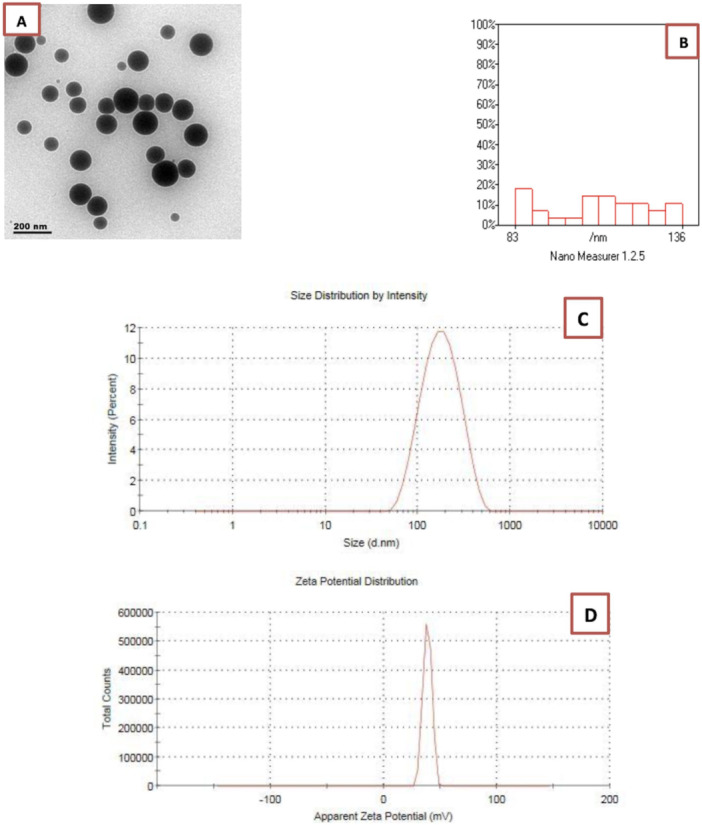
The morphology and size characteristics of rosmarinic acid‐loaded chitosan nanoparticles. (A) TEM analysis shows nearly spherical particles. (B) A histogram demonstrates that the majority of particles fall within 80–136 nm, indicating a narrow size distribution. Panels (C) and (D) display the volume‐based particle size distribution and zeta potential, respectively.

**Figure 2:**
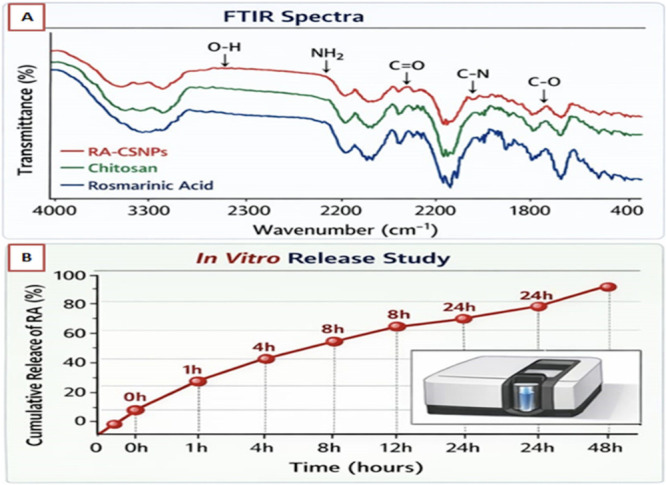
FTIR analysis and in vitro release behavior of RA‐CHNPs. (A) FTIR spectra illustrate the successful encapsulation of RA and the presence of non‐covalent interactions between RA and chitosan. (B) In vitro release profile of RA in PBS (pH 7.4, 37°C), showing an initial burst release followed by a sustained release pattern over 48 h.

Figure 1A illustrates that RA‐CHNPs exhibit well‐defined, spherical morphology when observed by TEM, with no signs of aggregation. The particle sizes ranged from 80 to 136 nm, indicating a uniform, consistent size distribution (Figure 1B). Dynamic light scattering (DLS) results indicated that RA‐CHNPs exhibited an average hydrodynamic diameter of 156 nm with a polydispersity index (PDI) of 0.172, showing that the particles were pretty uniform in size (Figure 1C). Figure 1D illustrates that RA‐CHNPs possessed a zeta potential of 38.8 mV, indicative of robust colloidal stability. The negative surface charge generates repulsive forces between particles, limiting aggregation and ensuring a well‐dispersed suspension. Additionally, the nanoparticles attained an encapsulation proficiency of 86.90% and a drug loading of 14.47%. FTIR analysis of crude RA, Chitosan, and RA‐CHNPs revealed notable modifications in their characteristic functional group signals (Figure 2A). The broad O–H stretching bands observed in RA and Chitosan shifted and decreased in intensity in the RA‐CHNPs spectrum, suggesting the formation of hydrogen bonds between RA and the Chitosan framework. The reduction of RA's characteristic peaks further demonstrates its incorporation into the Chitosan network rather than simple blending. Figure 2B shows that RA‐CHNPs release RA in a biphasic manner in PBS (pH 7.4). A moderate release at the early stages likely originates from RA molecules located near the nanoparticle surface, followed by a sustained release extending up to 48 h. This prolonged release indicates that RA is effectively encapsulated within the Chitosan–TPP crosslinked network, highlighting the substantial role of RA‐CHNPs as a stable and controlled delivery system under physiological conditions (Figure 2B).

### 3.2 Storage Stability of RA‐CHNPs Over Time

Table 2 demonstrates that RA‐CHNPs exhibit good storage stability over time. A slight, gradual increase in particle size is observed, remaining within an acceptable range and not indicating aggregation or formulation instability. In addition, the PDI values remained consistently below 0.2 throughout the storage period, indicating a highly uniform particle‐size distribution and the absence of significant aggregation. The zeta potential values also remained relatively high (above ±30 mV), with only a minor decrease over time, suggesting sustained electrostatic repulsion between particles and good colloidal stability.

**Table 2:** Stability of RA‐loaded chitosan nanoparticles (RA‐CHNPs) under storage conditions (4°C over 3 months).

Time point	Particle size (nm)	Polydispersity index (PDI)	Zeta potential (mV)
Day 0	156 ± 4.12	0.172 ± 0.01	38.8 ± 1.3
Month 1	157 ± 5.01	0.176 ± 0.02	37.6 ± 1.6
Month 2	161 ± 6.06	0.180 ± 0.02	37.3 ± 1.4
Month 3	164 ± 5.83	0.183 ± 0.02	36.4 ± 1.3

### 3.3 Effects of RA and RA‐CHNPs on Testicular Weight and Semen Quality

As indicated in Table 3, exposure to CFP led to a significant decrease in testicular weight to around 64.7% of the control group values (*p* < 0.0001). However, the co‐administration of RA‐CHNPs with CFP resulted in a notable increase in the testicular weight relative to CFP‐treated rats (*p* = 0.0001) with restoration of testicular weight to approximately 87.9% of the control group levels, with no significant difference from the negative control group (*p* = 0.11). In contrast, rats receiving both CFP and crude RA did not show a significant increase in testicular weight compared with those treated with CFP alone (*p* = 0.28). There was a significant difference between CFP + RA and CFP + RA‐CHNPs treated groups' testicular weight data sets (*p* = 0.0036).

**Table 3:** Effect of crude rosmarinic acid (RA) and RA‐loaded chitosan nanoparticles (RA‐CHNPs) on testicular weight and sperm quality in chlorfenapyr (CFP)‐exposed male rats.

Groups	Testes (g)	Sperm count (x10^6^)	Sperm motility (%)	Sperm abnormalities (%)
Control	1.62 ± 0.08^**ab**^	64.28 ± 3.16^**a**^	81.69 ± 4.15^**a**^	5.15 ± 1.02^**c**^
RA	1.67 ± 0.07^**a**^	65.19 ± 2.84^**a**^	82.46 ± 5.21^**a**^	4.74 ± 0.84^**c**^
RA‐CHNPs	1.68 ± 0.09^**a**^	66.37 ± 4.15^**a**^	82.71 ± 3.97^**a**^	4.53 ± 1.13^**c**^
CFP	1.13 ± 0.01^**c**^	22.06 ± 2.15^**c**^	42.26 ± 3.02^**b**^	13.39 ± 1.25^**a**^
CFP + RA	1.24 ± 0.04^**c**^	31.29 ± 3.84^**bc**^	51.21 ± 2.44^**b**^	8.16 ± 1.48^**b**^
CFP + RA‐CHNPs	1.48 ± 0.03^**b**^	42.61 ± 3.06^**b**^	73.11 ± 3.62^**a**^	7.59 ± 0.77^**bc**^
*p*‐values	< 0.0001	< 0.0001	0.0001	0.0005

*Note:* Results are expressed as ± SE, and values with different superscripts (a, b, c) within the same row differ significantly at *p* < 0.05.

Abbreviations: CFP, chlorfenapyr (180 mg/kg body weight); CFP/RA, rosmarinic acid (75 mg/kg) + chlorfenapyr (180 mg/kg); CFP/RA‐CHNPs, RA‐loaded chitosan nanoparticles (75 mg/kg) + chlorfenapyr (180 mg/kg); RA, rosmarinic acid (75 mg/kg body weight); RA‐CHNPs, rosmarinic acid‐loaded chitosan nanoparticles (75 mg/kg body weight).

In terms of semen quality, CFP exposure resulted in a significant decline in both sperm count and motility (approximately to 33.7% and 50.8% of the control group values; *p* < 0.0001) and increased the percentage of abnormal sperm compared with all control groups (2.8‐fold rise, *p* < 0.0001). Co‐treatment with RA‐CHNPs led to a significant rise in both sperm count and motility (1.9‐ and 1.7‐fold rise, respectively *p* < 0.0001) and concurrent decline in the percentage of abnormal sperm compared with the CFP group (Around 44.6% decline; *p* < 0.0001), bringing sperm motility and abnormality percentages back to levels similar to the negative control (*p* > 0.05). Meanwhile, treatment with crude RA alongside CFP only partially improved the sperm motility as well as percentage of abnormal sperm compared to CFP group (*p* = 0.03 and 0.001, respectively). Interestingly, there were significant differences between CFP + RA and CFP + RA‐CHNPs treated groups' semen the sperm count and motility (*p* = 0.0036, and 0.0003) with no notable differences in the percentage of abnormal sperm data sets (*p* = 0.98).

### 3.4 Effects of RA and RA‐CHNPs on Reproductive Hormones

**Figure 3:**
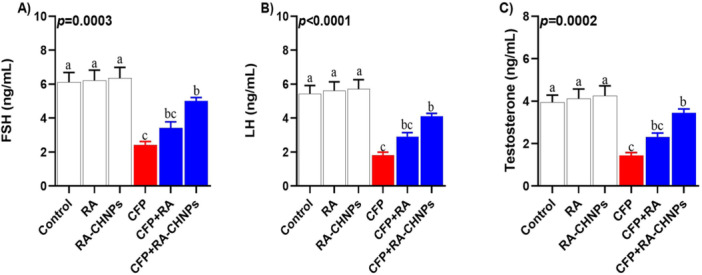
Effect of crude rosmarinic acid (RA) and rosmarinic acid–loaded chitosan nanoparticles (RA‐CHNPs) on reproductive hormone levels in male rats exposed to chlorfenapyr (CFP). (A): FSH refers to follicle‐stimulating hormone, (B): LH refers to luteinizing hormone, and (C): refers to testosterone hormone. Experimental groups included: RA (rosmarinic acid, 75 mg/kg body weight); RA‐CHNPs (rosmarinic acid–loaded chitosan nanoparticles, 75 mg/kg body weight); CFP (chlorfenapyr, 180 mg/kg body weight); CFP/RA (rosmarinic acid, 75 mg/kg + chlorfenapyr, 180 mg/kg); and CFP/RA‐CHNPs (rosmarinic acid–loaded chitosan nanoparticles, 75 mg/kg + chlorfenapyr, 180 mg/kg). Data are presented as mean ± standard error (SE). Values bearing different superscript letters (a, b, c) within the same row indicate statistically significant differences at *p* < 0.05.

Exposure of male rats to CFP led to a significant decrease in the level of serum FSH (Figure 3A), LH (Figure 3B), and testosterone hormone (Figure 3C) to around 38.9%, 32.8%, and 35.2% of control group levels (*p* < 0.0001), demonstrating a marked disturbance in the hypothalamic–hypophyseal–gonadal axis. Administration of RA‐CHNPs significantly ameliorated CFP‐induced hormonal disturbances, as evidenced by significantly increased serum FSH, LH, and testosterone levels compared with the CFP‐treated group (*p* = 0.02, 0.01, and 0.008, respectively). In contrast, co‐administration of crude RA did not result in statistically significant improvements in tested hormones levels compared with the CFP‐treated group (*p* > 0.05).

### 3.5 Effects of RA and RA‐CHNPs on Oxidative Stress and NRF2/HO‐1 Pathway

**Figure 4:**
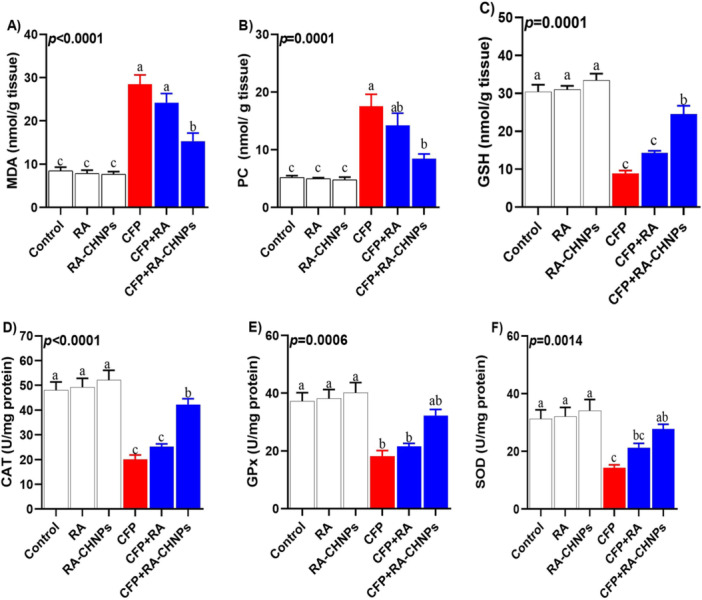
Effect of crude rosmarinic acid (RA) and rosmarinic acid–loaded chitosan nanoparticles (RA‐CHNPs) on oxidative stress parameters in testicular tissues of male rats exposed to chlorfenapyr (CFP). (A) Malondialdehyde (MDA), (B) protein carbonyls (PC), (C) reduced glutathione (GSH), (D) catalase (CAT), (E) glutathione peroxidase (GPx), and (F) superoxide dismutase (SOD) were assessed. Experimental groups included: RA (rosmarinic acid, 75 mg/kg body weight); RA‐CHNPs (rosmarinic acid–loaded chitosan nanoparticles, 75 mg/kg body weight); CFP (chlorfenapyr, 180 mg/kg body weight); CFP/RA (rosmarinic acid, 75 mg/kg + chlorfenapyr, 180 mg/kg); and CFP/RA‐CHNPs (rosmarinic acid–loaded chitosan nanoparticles, 75 mg/kg + chlorfenapyr, 180 mg/kg). Data are presented as mean ± standard error (SE). Values bearing different superscript letters (a, b, c) within the same row indicate statistically significant differences at *p* < 0.05.

Exposure to CFP led to a significant increase in oxidative stress biomarkers, as evidenced by elevated levels of the lipid peroxidation markers MDA (Figure 4A) and PC (Figure 4B) compared with all control groups (approximately 3.5‐ and 3.2‐fold rise, respectively; *p* < 0.0001). Co‐administration of RA‐CHNPs significantly reduced these elevations to around 54.7% and 47.2%, respectively, of CFP group levels (*p* < 0.0001; for both), while insignificant differences were seen between the CFP‐exposed group and the group receiving crude RA (*p* > 0.05).

Regarding the antioxidant defense system, exposure to CFP resulted in a marked reduction of GSH content (Figure 4C) and a significant decline in the activities of critical antioxidant enzymes, including CAT (Figure 4D), GPx (Figure 4E), and SOD (Figure 4F), in comparison to all control groups (around 71.3%, 58.2%, 52.1%, and 55.2% decline, respectively; *p* < 0.0001 for all). Conversely, co‐treatment with RA‐CHNPs significantly restored GSH levels (*p* < 0.0001) and improved CAT (*p* = 0.007), GPx (*p* = 0.03) and SOD (*p* = 0.02) activities beyond those observed with the CPF group levels. Additionally, GPx and SOD activities in the CFP + RA‐CHNPs‐treated group were similar to those in the negative control group (*p* > 0.05). There were significant differences between CFP + RA and CFP + RA‐CHNPs treated groups' GSH and CAT data sets (*p* = 0.0036 and 0.01, respectively).

**Figure 5:**
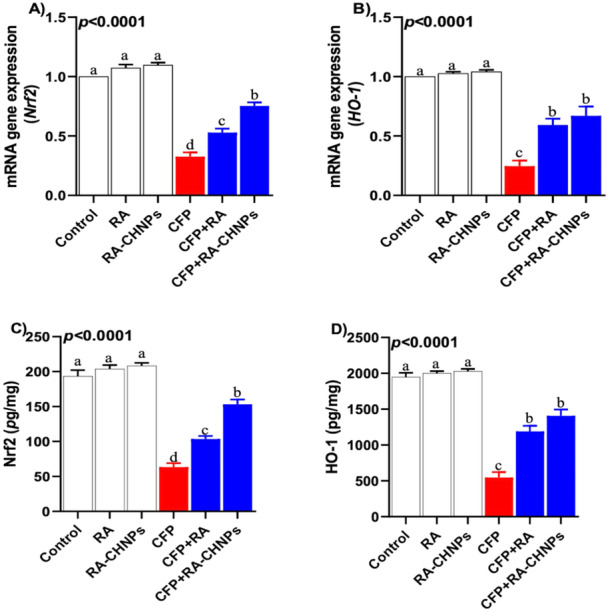
Effect of crude rosmarinic acid (RA) and rosmarinic acid–loaded chitosan nanoparticles (RA‐CHNPs) on the NRF2/HO‐1 pathway in testicular tissues of male rats exposed to chlorfenapyr (CFP). (A) [Nuclear factor erythroid 2–related factor 2 (*Nrf2*)], (B) [Heme Oxygenase‐1 (*HO‐1*)], (C) (*Nrf2*) and (D) (*HO‐1*). Experimental groups included: RA (rosmarinic acid, 75 mg/kg body weight); RA‐CHNPs (rosmarinic acid–loaded chitosan nanoparticles, 75 mg/kg body weight); CFP (chlorfenapyr, 180 mg/kg body weight); CFP/RA (rosmarinic acid, 75 mg/kg + chlorfenapyr, 180 mg/kg); and CFP/RA‐CHNPs (rosmarinic acid–loaded chitosan nanoparticles, 75 mg/kg + chlorfenapyr, 180 mg/kg). Data are presented as mean ± standard error (SE). Values bearing different superscript letters (a, b, c) within the same row indicate statistically significant differences at *p* < 0.05.

At the molecular level, exposure to CFP significantly repressed the Nrf2/HO‐1 signaling pathway compared with all control groups. Exposure to CFP significantly downregulated Nrf2 and HO‐1genes expression levels to 32.4% and 24.7%, respectively, of the control group levels (*p* < 0.001; Figure 5A,B) with concomitant decline in their corresponding proteins (*p* < 0.0001; Figure 5C,D). Co‐administration of CFP with either RA formulation significantly upregulated Nrf2 and HO‐1 gene expression compared to the CFP‐treated group (*p* < 0.0001, and 0.0003, respectively) and their protein levels (*p* < 0.0001; for both proteins). Notably, co‐treatment with RA‐CHNPs induced a significantly greater upregulation of Nrf2 expression and protein levels than co‐treatment with crude RA (*p* = 0.003, and 0.001, respectively), whereas there was no significant difference between the two formulations with respect to HO‐1 expression and protein levels (*p* > 0.05).

### 3.6 Effects of RA and RA‐CHNPs on Inflammatory Response and NF‐κB/NLRP3 Inflammasome Pathways

**Figure 6:**
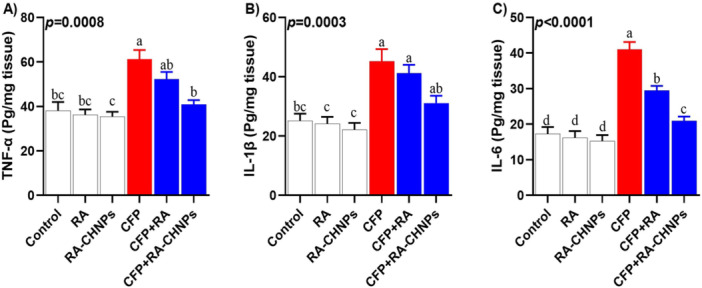
Effect of crude rosmarinic acid (RA) and rosmarinic acid–loaded chitosan nanoparticles (RA‐CHNPs) on inflammatory responses in testicular tissues of male rats exposed to chlorfenapyr (CFP). (A) Tumor necrosis factor‐alpha (TNF‐α), (B) Interleukin‐1 beta (IL‐1β), and (C) Interleukin‐6 (IL‐6). Experimental groups included: RA (rosmarinic acid, 75 mg/kg body weight); RA‐CHNPs (rosmarinic acid–loaded chitosan nanoparticles, 75 mg/kg body weight); CFP (chlorfenapyr, 180 mg/kg body weight); CFP/RA (rosmarinic acid, 75 mg/kg + chlorfenapyr, 180 mg/kg); and CFP/RA‐CHNPs (rosmarinic acid–loaded chitosan nanoparticles, 75 mg/kg + chlorfenapyr, 180 mg/kg). Data are presented as mean ± standard error (SE). Values bearing different superscript letters (a, b, c) within the same row indicate statistically significant differences at *p* < 0.05.

In terms of the inflammatory response, CFP exposure significantly increased pro‐inflammatory cytokines, including TNF‐α (Figure 6A), IL‐1β (Figure 6B), and IL‐6 (Figure 6C), compared with all control groups (1.6, 1.8, and 2.4‐fold rise, respectively; *p* < 0.0001). Co‐administration of RA‐CHNPs with CFP significantly reduced TNF‐α and IL‐1β levels to approximately 67.2%, 70.2%, and 54.7% of CFP group values, respectively (*p* = 0.005, 0.03, and < 0.0001, respectively), bringing them back to levels comparable to those of the negative control group (*p* > 0.05). Regarding crude RA treatment, IL‐6 declined significantly in comparison with CPF‐treated group (*p* = 0.0044). In contrast, there was no significant difference between the CFP‐exposed group and the crude RA group in TNF‐α and IL‐1β levels (*p* > 0.05). There were significant differences between CFP + RA and CFP + RA‐CHNPs treated groups' IL‐6 data sets (*p* = 0.03).

**Figure 7:**
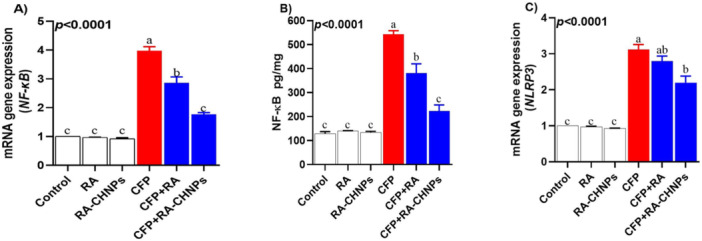
Effect of crude rosmarinic acid (RA) and rosmarinic acid–loaded chitosan nanoparticles (RA‐CHNPs) on NF‐κB/NLRP3 inflammasome signaling pathways in testicular tissues of male rats exposed to chlorfenapyr (CFP). (A) [Nuclear Factor kappa B (NF‐κB)], (B) (NF‐κB) and (C) [NOD‐like receptor family pyrin domain‐containing 3 (NLRP3)]. Experimental groups included: RA (rosmarinic acid, 75 mg/kg body weight); RA‐CHNPs (rosmarinic acid–loaded chitosan nanoparticles, 75 mg/kg body weight); CFP (chlorfenapyr, 180 mg/kg body weight); CFP/RA (rosmarinic acid, 75 mg/kg + chlorfenapyr, 180 mg/kg); and CFP/RA‐CHNPs (rosmarinic acid–loaded chitosan nanoparticles, 75 mg/kg + chlorfenapyr, 180 mg/kg). Data are presented as mean ± standard error (SE). Values bearing different superscript letters (a, b, c) within the same row indicate statistically significant differences at *p* < 0.05.

At the molecular level, CFP exposure significantly activated the NF‐κB/NLRP3 inflammasome signaling pathways (Figure 7A–C) compared with all control groups. CFP exposure has significantly raised NF‐κB gene expression and protein levels as well as NLRP3 gene expression levels to around 3.2, 4.4, and 3.1‐fold, respectively, of the control group levels (*p* < 0.0001). Conversely, co‐administration of RA‐CHNPs with CFP significantly reduced NF‐κB and NLRP3 gene expression and NF‐κB protein levels in comparison with the CFP‐only treated group (*p* < 0.0001; for all), with NF‐κB expression returning to levels not significantly different from those in the negative control group (*p* > 0.05). Additionally, there was no significant difference in NLRP3 gene expression between the CFP + crude RA‐treated group and the control group (*p* = 0.35). There were significant differences between CFP + RA and CFP + RA‐CHNPs treated groups' NF‐κB gene expression and protein levels, as well as NLRP3 gene expression levels data sets (*p* < 0.0001, 0.0013, and 0.02, respectively).

### 3.7 Effects of RA and RA‐CHNPs on Apoptosis‐Related Gene Expression

**Figure 8:**
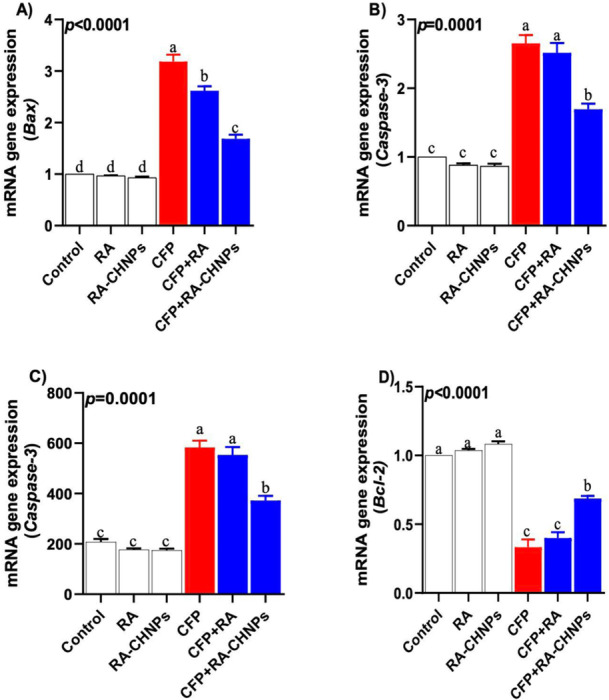
Effect of crude rosmarinic acid (RA) and rosmarinic acid–loaded chitosan nanoparticles (RA‐CHNPs) on NF‐κB/NLRP3 inflammasome signaling pathways in testicular tissues of male rats exposed to chlorfenapyr (CFP). (A) Bcl‐2–associated X protein (*Bax*), (B, C) Cysteine‐aspartic acid protease‐3 (*Caspase‐3*), and (D) B‐cell lymphoma 2 (BCL‐2). Experimental groups included: RA (rosmarinic acid, 75 mg/kg body weight); RA‐CHNPs (rosmarinic acid–loaded chitosan nanoparticles, 75 mg/kg body weight); CFP (chlorfenapyr, 180 mg/kg body weight); CFP/RA (rosmarinic acid, 75 mg/kg + chlorfenapyr, 180 mg/kg); and CFP/RA‐CHNPs (rosmarinic acid–loaded chitosan nanoparticles, 75 mg/kg + chlorfenapyr, 180 mg/kg). Data are presented as mean ± standard error (SE). Values bearing different superscript letters (a, b, c) within the same row indicate statistically significant differences at *p* < 0.05.

Exposure to CFP significantly altered the expression of pro‐apoptotic genes, such as Bax (Figure 8A) and Caspase‐3 (Figure 8B), compared with all control groups (3.2 and 2.6‐fold rise, respectively; *p* < 0.0001). Co‐administration of RA‐CHNPs alongside CFP decreased the expression levels of Bax and Caspase‐3 significantly to around 53.1% and 65.7% of CPF group values (*p* < 0.0001; for both), demonstrating a significantly stronger effect than that of crude RA co‐treatment (*p* < 0.0001, and 0.0003, for Bax and caspase‐3, respectively). Importantly, there was no significant difference in Caspase‐3 expression between the CFP‐exposed group and the RA‐CHNPs + CFP group (*p* = 0.99). This trend was corroborated by the results from the ELISA assay for caspase‐3 expression, which aligned with the changes in the Caspase‐3 coding gene expression (Figure 8C). Conversely, CFP exposure also significantly altered the expression of the anti‐apoptotic gene *Bcl‐2* to around 32.8% of the control group Bcl‐2 expression levels (*p* < 0.0001) (Figure 8D). Co‐treatment with RA‐CHNPs resulted in a significant increase in *Bcl‐2* expression compared to CFP‐treated group (*p* < 0.0001), showing greater restoration compared to crude RA co‐treatment, which did not show any significant variations from the CFP‐exposed group (*p* = 0.65).

### 3.8 Effects of RA and RA‐CHNPs on Histopathological Changes

**Figure 9:**
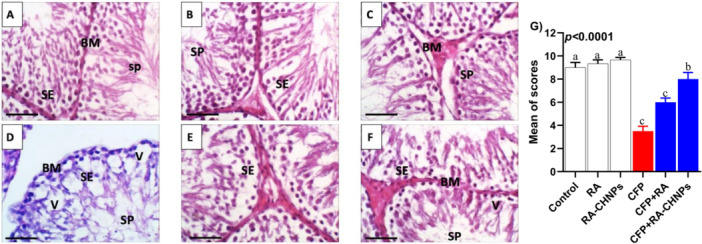
Representative photomicrographs of testes from control and experimental groups: (A) Control; (B) RA; (C) RA‐CHNPs; (D) CFP; (E) CFP + AL; (F) CFP + RA‐CHNPs; (G) Mean of scores; BM = basement membrane, SP = spermatozoa, SE = seminiferous epithelium. Images were captured at 400× magnification; scale bar = 50 µm.

Figure 9 illustrates the histological features of testicular tissue in the CFP‐exposed rats, and the alterations observed following treatment with either crude RA or RA‐CHNPs. Figure 9A–C depict that in the controls (RA‐treated, and RA‐CHNPs‐treated groups) the spermatogenic cells appeared intact, and the overall arrangement of the spermatogenic layers was well‐maintained. In the CFP‐treated group, testicular tissue displayed marked structural damage, characterized by extensive detachment of germinal epithelial cells. Frequent nuclear changes, including karyorrhexis and pyknosis, were observed, indicating significant tissue injury induced by CFP exposure (Figure 9D). In comparison with the CFP group, testicular sections from the CFP + RA and CFP + RA‐CHNPs groups largely maintained normal spermatocyte structures, showing only minor changes such as slight thinning of the spermatogenic layers and occasional intracellular vacuoles. Overall tissue architecture remained well preserved, and the germinal epithelium supported normal germ cell development (Figures 9E and 9F). Johnsen's scoring system for spermatogenesis evaluation revealed that CFP rats demonstrated a significantly lower mean score of 3.5 ± 1.1, compared to the control score of 9 ± 0.9 (*p* < 0.0001). This score was significantly improved in CFP + RA (6 ± 0.89) and CFP + RA‐CHNPs (8 ± 1.4) groups. While RA‐CHNPs resulted in a more significant recovery (*p* < 0.016), demonstrating the enhanced protective effect of the nanoliposomal formulation on testicular architecture and germ cell maturation (Figure 9G).

### 3.9 Immunohistochemical Expression of NF‐κB and NLRP3 in Testicular Tissue

**Figure 10:**
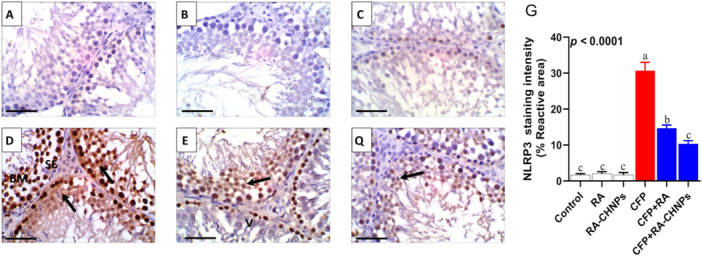
A representative testicular immunohistochemical staining for NF‐κB from control and experimental groups: (A) Control; (B) RA; (C) RA‐CHNPs; (D) CFP; (E) CFP + AL; (F) CFP + RA‐CHNPs. Minimal and diffuse expression was observed in the control, RA, and RA‐CHNPs groups. The CFP group exhibited strong and widespread NF‐κB expression in spermatogenic and interstitial Leydig cells, with cytoplasmic and nuclear staining in spermatocytes (arrow) and high expression in round spermatids. Treatment with CFP + AL or CFP + RA‐CHNPs led to a moderate reduction of NF‐κB expression in seminiferous tubules, with few positive Leydig cells, scant expression in spermatogonia, reduced cytoplasmic staining in spermatocytes, and increased expression in round spermatids without nuclear localization (arrow). All images were captured at 400× magnification, scale bar 50 µm. (G) Scores of NF‐κB immunohistochemical across the experimental groups. Data are presented as mean ± standard error (SE). Values bearing different superscript letters (a, b, c) within the same row indicate statistically significant differences at *p* < 0.05.

**Figure 11:**
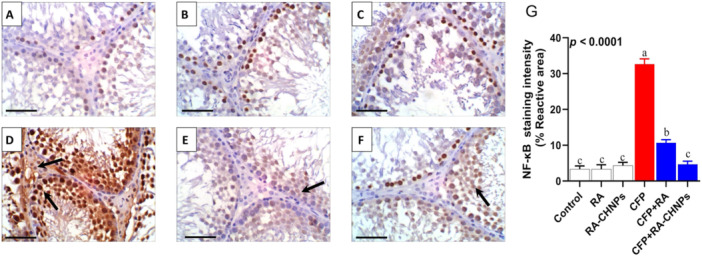
A representative photomicrograph of testicular immunohistochemical staining for NLRP3 in control and experimental groups. (A) Control; (B) RA; (C) RA‐CHNPs; (D) CFP; (E) CFP + AL; (Q) CFP + RA‐CHNPs. The control, RA, and RA‐CHNPs groups showed negative NLRP3 expression in spermatogenic cells. In contrast, the CFP group exhibited strong positive NLRP3 expression in spermatogenic cells (arrow). Treatment with CFP + AL or CFP + RA‐CHNPs resulted in a moderate reduction of NLRP3 expression within the seminiferous tubules (arrow). All images were captured at 400× magnification, with a scale bar of 50 µm. (G) Scores of NLRP3 immunohistochemical across the experimental groups. Data are presented as mean ± standard error (SE). Values bearing different superscript letters (a, b, c) within the same row indicate statistically significant differences at *p* < 0.05.

Immunohistochemical analysis of testicular tissue revealed distinct patterns of NF‐κB and NLRP3 expression among the experimental groups. In the RA and RA‐CHNPs control groups, NF‐κB expression in spermatogenic cells was minimal and diffuse (scored 3.3 ± 1.5). In contrast, the CFP group showed markedly elevated NF‐κB expression (Scored 32.6 ± 2.5) in spermatogenic cells and interstitial Leydig cells, among spermatocytes, cytoplasmic and nuclear staining, and particularly high expression in round spermatids. Treatment with CFP + AL or CFP + RA‐CHNPs resulted in a moderate significant (*p* < 0.0001; for both relative to CFP‐treated group) reduction of NF‐κB expression (Scored 10.6 ± 1.4 and 4.5 ± 1.7, respectively) within the seminiferous tubules, with limited positive Leydig cells, scant expression in spermatogonia, reduced cytoplasmic staining in spermatocytes, and increased expression in round spermatids without nuclear localization. There was a significant difference between RA and RA‐CHNPs treated groups in NF‐κB immno‐scores (*p* = 0.02) (Figure 10). Similarly, NLRP3 expression was absent in control (Scored 1.6 ± 0.6), RA (Scored 2.1 ± 0.9), and RA‐CHNPs (Scored 1.6 ± 1.1) groups, strongly positive in the CFP group (Scored 30.6 ± 3.9), and moderately declined in the groups treated with CFP + AL (Scored 16.2 ± 1.1) and CFP + RA‐CHNPs (Scored 9 ± 0.9). There was a significant difference between RA and RA‐CHNPs treated groups in NLRP3 immno‐scores (*p* = 0.006) (Figure 11).

### 3.10 Multivariate Analysis

**Figure 12:**
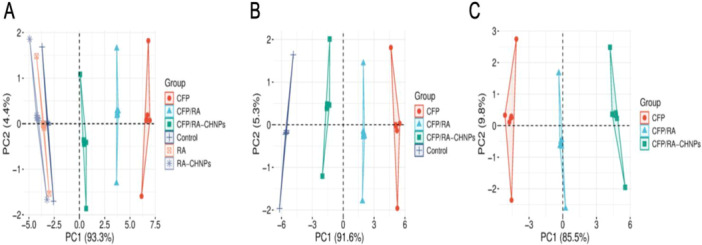
Principal component analysis (PCA) illustrating the multivariate effects of chlorfenapyr (CFP) and the protective role of rosmarinic acid (RA) and nano‐rosmarinic acid–loaded chitosan nanoparticles (RA‐CHNPs) on the studied parameters in male rats. (A–C) PCA score plots showing the distribution and clustering of experimental groups along the first two principal components (PC1 and PC2), with the percentage of variance explained by each component indicated on the axes. Each point represents an individual animal, and dashed lines indicate the origin of the PCA axes.

Principle component analysis was performed to consolidate and illustrate the multivariate correlations of parameters across the experimental groups. Figure 12A–C demonstrates that PCA effectively and consistently distinguished the CFP‐treated group from the control group along the first principal component (PC1). This represented the majority of the total variance (ranging from 85.5% to 93.3%), indicating that CFP exposure resulted in significant global disruption. The CFP group constituted a distinct cluster associated with adverse outcomes. In contrast, rats treated with RA or RA‐CHNPs showed a close clustering with the control group, suggesting that physiological homeostasis was preserved. Co‐administration of RA‐CHNPs induced a significant shift in the CFP‐exposed samples towards the control cluster. This indicates that RA‐CHNPs exhibited a more pronounced corrective effect compared to crude RA.

## 4 Discussion

Pesticide‐related reproductive toxicity has emerged as a critical risk factor for environmental safety. CFP, extensively used as a pyrrole insecticide, has increasingly been associated with testicular toxicity [4, 5]. Excessive generation of reactive oxygen species is a key mechanism underlying CFP‐mediated cellular injury and compromises steroid hormone synthesis and spermatogenesis [44]. Understanding the underlying mechanisms is fundamental to advancing therapeutic approaches that preserve male reproductive health. The present study hypothesized that RA‐CHNPs could mitigate CFP‐induced toxicity by attenuating oxidative stress, reducing inflammation and apoptosis, and maintaining hormonal function and spermatogenesis, thereby enhancing semen quality and normalizing testosterone levels compared with crude RA.

Exposure to CFP in this study was associated with significant testicular atrophy and severe deterioration in sperm count, motility, and viability, as well as marked suppression of circulating levels of testosterone, LH, and FSH. These observations highlight severe dysfunction in both hormonal output and sperm production, supporting prior evidence that pesticides induce testicular toxicity by impairing androgen biosynthesis and disrupting sperm parameters [5, 45]. According to Hassan et al., a decline in testicular weight is commonly accompanied by germ cell loss and seminiferous tubule degeneration [46].

Additionally, the observed impairments in sperm count, motility, and viability reflect underlying germ cell damage, presumably mediated by DNA fragmentation, mitochondrial disruption, and oxidative membrane injury [19, 47]. The decline in testosterone concentrations also suggests Leydig cell impairment, possibly due to oxidative stress and inflammatory signaling that interfere with hormonal regulation and steroidogenic enzyme activity [48]. Importantly, administration of RA‐CHNPs was significantly associated with the mitigation of these adverse reproductive effects more effectively than crude RA. Treatment with RA‐CHNPs was associated with restored testicular weight, improved sperm parameters, and normalization of reproductive hormone levels. The observed enhancements highlight the potential protective role of RA‐CHNPs in sustaining endocrine activity and spermatogenesis, primarily by mitigating oxidative stress, suppressing inflammatory response, and safeguarding mitochondrial and cellular structures [49]. Restoration of LH and FSH levels further suggests that reducing oxidative stress may help re‐establish proper pituitary–gonadal axis activity. These observations are consistent with previous studies exhibiting significant improvements in sperm parameters in rats treated with RA after pesticide or xenobiotic‐induced reproductive toxicity.

According to Şahin et al., RA mitigated cyclophosphamide‐induced gonadal toxicity and enhanced reproductive function in adult rats [13]. Also, Norman et al. found that RA protected against cisplatin‐induced testicular damage in mice by modulating oxidative stress, inflammation, and apoptosis, and by activating the Nrf2/HO‐1 signaling pathway [10]. Likewise, Abduh et al. found that RA modulated chlorpyrifos‐induced oxidative stress, inflammation, and kidney damage by reducing oxidative stress and restoring SIRT1 and Nrf2/HO‐1 pathway activity [12].

This study provides evidence that oxidative stress is a major contributor to CFP‐induced testicular toxicity. Overproduction of ROS following CFP exposure exceeds endogenous antioxidant defenses, leading to oxidative injury to nucleic acids, proteins, and lipids and eventually compromising testicular function and integrity [6, 9]. The current observations are consistent with prior reports, suggesting that overproduction of ROS may induce testicular injury [50]. Also, CFP‐induced toxicity involves mitochondrial dysfunction, as it interferes with oxidative phosphorylation, positioning mitochondria as both a primary ROS generator and a vulnerable target [6, 51].

Mitochondrial DNA is highly vulnerable to oxidative injury owing to limited repair mechanisms and its proximity to the electron transport chain [52]. This irreversible damage further increases ROS production, resulting in a self‐reinforcing cycle of oxidative injury and mitochondrial dysfunction that ultimately leads to testicular degeneration and the apoptosis of germ cells [53, 54]. Consistent with this mechanism, the current study identified significant increases in MDA and PC levels, along with notable decreases in GSH content and the activities of key antioxidant enzymes, including SOD, GPX, and CAT. These findings correspond with previous research on testicular oxidative damage caused by pesticides or drugs, emphasizing lipid peroxidation as a critical outcome of oxidative stress that disrupts membrane fluidity and protein functionality, resulting in cell death [4, 55, 56, 57]. Additionally, oxidative modifications of proteins, including aggregation, carbonylation, and fragmentation, further impair intracellular signaling and enzyme function, thereby exacerbating testicular injury [58].

Indeed, the present results demonstrate that administration of RA‐CHNPs was associated with potent antioxidative effects against CFP‐induced testicular oxidative stress. RA‐CHNPs administration significantly suppressed ROS overproduction, as reflected by decreased MDA and PC levels and the restoration of GSH, SOD, and CAT, more effectively than crude RA. The observed protective effects are mainly due to RA's potent antioxidant activity, suggesting improvement in the bioavailability and stability afforded by Chitosan nanoencapsulation [49, 59]. By the noticed associated decreased oxidative damage to cellular components and safeguarding mitochondrial function, RA‐CHNPs broke the cycle of ROS overproduction and mitochondrial dysfunction, supporting normal sperm production and testicular integrity [20, 60]. Moreover, the protective effects of RA‐CHNPs were closely associated with activation of the Nrf2/HO‐1 signaling pathway [10]. Indeed, the activation of Nrf2 improves cellular antioxidant and anti‐inflammatory defenses while concurrently suppressing NF‐κB–mediated inflammatory signaling by stabilizing IκB‐α [10, 61, 62]. We observed that CFP exposure significantly downregulated Nrf2 and HO‐1 in the testes, whereas RA‐CHNP administration was associated with reinstatement of Nrf2/HO‐1 pathway activity. This aligns with prior reports showing RA protects against xenobiotic‐induced testicular and hepatorenal damage via Nrf2‐mediated potential cytoprotective actions [10, 63].

Chlorfenapyr‐induced testicular injury involves not only oxidative stress but also inflammatory signaling, in which excessive ROS generation activates NF‐κB/NLRP3 inflammasome pathways, promoting the production of pro‐inflammatory cytokines such as TNF‐α, IL‐1β, and IL‐6 [64, 65, 66]. The interaction between inflammation and oxidative stress establishes a self‐reinforcing cycle that aggravates testicular injury [50, 67]. An intra‐testicular elevation of NF‐κB, NLRP3, and pro‐inflammatory cytokines has been repeatedly observed in chemically induced testicular toxicity models, reflecting the central role of inflammation in reproductive impairment [68, 69, 70]. Testicular inflammation disrupts spermatogenesis by impairing steroidogenic activity, reducing Leydig cell testosterone synthesis, and interfering with germ cell proliferation and differentiation, thereby reducing male fertility [71, 72]. Furthermore, persistent oxidative stress and inflammation synergistically trigger intrinsic apoptosis, as indicated by upregulation of Bax, downregulation of Bcl‐2, and activation of caspase‐3, leading to germ cell death [50, 73, 74]. Mechanistically, excessive production of ROS compromises the integrity of the mitochondrial membrane, promoting the release of cytochrome c and activating the caspase‐dependent apoptotic pathway, especially caspase‐3 [75]. This cascade leads to structural degeneration of testicular tissue, compromised spermatogenesis, and subsequent male infertility [76, 77].

The sensor NLRP3, the adaptor ASC, and pro‐caspase‐1 make up the NLRP3 inflammasome, a cytosolic multiprotein complex that, when activated, assembles and auto‐cleaves caspase‐1. The maturation and production of IL‐1β and IL‐18 are subsequently encouraged by activated caspase‐1, which intensifies the local inflammatory reactions [78]. Crucially, upstream signals, including ROS overproduction and NF‐κB‐mediated priming, which trigger the transcription of NLRP3 and pro‐IL‐1β, tightly regulate NLRP3 activity [79]. Persistent NLRP3 activation in testicular tissue directly contributes to spermatogenic failure via encouraging blood–testis barrier rupture, Sertoli cell dysfunction, and accelerated germ cell loss [69]. However, it should be noted that the current study evaluated NLRP3 expression at the gene level, so definitive confirmation of inflammasome activation would require assessment of downstream effectors, including caspase‐1 cleavage and mature IL‐18 production, which were not examined in the present study.

Effective mitigation of pesticide‐induced testicular damage via inflammation suppression further underscores the critical interplay between oxidative stress, inflammatory signaling, and programmed cell death in testicular dysfunction [80]. In this study, RA‐CHNP administration was significantly associated with attenuated CFP‐induced inflammation and apoptosis in the testes. Treatment significantly inhibited NF‐κB/NLRP3 pathway activation and reduced testicular tissue levels of TNF‐α, IL‐1β, and IL‐6 levels, more effectively than crude RA. At the same time, apoptotic signaling was modulated by restoring Bcl‐2 levels while downregulating caspase‐3 and Bax expression, indicating effective suppression of mitochondria‐dependent apoptosis.

Although there were no direct‐comparison studies, the present results align with earlier studies showing that RA induces anti‐inflammatory and anti‐apoptotic effects throughout NF‐κB suppression across different pathological contexts [81, 82]. In addition, Abduh et al. demonstrated that RA mitigates CFP‐induced kidney damage by reducing oxidative stress, inflammation, and apoptosis. Activating SIRT1 and Nrf2/HO‐1 pathways are other involved mechanisms [12]. Also, Fatahi et al. reported that RA alleviated radiation‐induced oxidative damage in the testes and restored antioxidant enzyme activities, including CAT, GPx, and SOD, resulting in significant improvements in histopathological markers and tissue integrity [56]. Histopathological analysis demonstrated that RA‐CHNP treatment maintained seminiferous tubule architecture, preserved germ cell viability, and protected Sertoli and Leydig cells from CFP‐induced damage. The enhanced efficacy of RA‐CHNP compared to crude RA suggests that nanoparticle encapsulation improves RA's bioavailability, stability, and targeted delivery, resulting in greater histological, biochemical, and functional outcomes. Overall, these results indicate that RA‐CHNP potentiate RA's protective impacts against oxidative and inflammatory testicular damage, thereby supporting reproductive and endocrine health.

The current study highlighted the role of Nrf2/HO‐1 and NF‐κB/NLRP3 in CFP‐induced marked oxidative stress and inflammatory responses, as evidenced by increased lipid peroxidation and activation of pro‐inflammatory signaling pathways. Also, both pathways showed a significant role in RA‐CHNPs' suggested protective effect. Interestingly, this dual effect is mechanistically interconnected, as activation of the Nrf2/HO‐1 pathway reduces intracellular ROS levels, which are key upstream triggers of NF‐κB signaling pathway activation and NLRP3 inflammasome assembly [83, 84]. Moreover, HO‐1–derived metabolites exert direct inhibitory effects on NF‐κB activation and inflammasome priming [85]. Thus, suppression of the NF‐κB/NLRP3 pathway by RA‐CHNPs is, at least in part, a downstream consequence of enhanced Nrf2‐mediated antioxidant signaling, highlighting a tightly regulated crosstalk between oxidative stress and inflammation.

Several limitations should be noted, despite it offering significant mechanistic and therapeutic insights. First, the study employed an oral relatively high dosage of CFP (1/3 LD50), which might not represent chronic low‐dose human exposure scenarios but is appropriate for evaluating diverse toxicological and mechanistic responses within a specific time frame. Second, the lack of Western blot analysis and immunohistochemistry (IHC) limited the confirmation of significant signaling pathways at the protein level, especially for major inflammasome constituents like caspase‐1 and IL‐1β, as well as NRF2 and NF‐κB pathway markers, even though oxidative stress, inflammation, and apoptosis were well assessed using biochemical assays, RT‐qPCR, and ELISA. In addition, although NLRP3 expression was evaluated at the gene and protein levels, definitive confirmation of inflammasome activation would require assessment of downstream effectors, including caspase‐1 cleavage and mature IL‐18 production, which were not examined in the present study. Third, accurate association between nanoformulation and improved biological efficacy is hampered by the lack of direct evaluation of the pharmacokinetic parameters of RA‐CHNPs, including tissue distribution and bioavailability. Also, the study was designed to compare crude RA and RA‐loaded CHNPs, an empty CHNP control was not investigated. Moreover, sex‐specific variations and their translational significance for human reproductive physiology require additional validation, as the study was limited to male rats. Lastly, no research was conducted on the long‐term safety or potential cumulative effects of repeated administration of the nanoparticles. To improve translational applicability, more research utilizing protein‐level validation, comprehensive pharmacokinetics, and lower, ecologically relevant dosage regimens is necessary.

## 5 Conclusion

This study confirms that CFP induces marked testicular toxicity attributed to oxidative stress, inflammation, hormonal disruption, and apoptosis. Encapsulation of RA in Chitosan nanoparticles significantly enhanced its protective efficacy compared with crude RA, which was associated with notable improvement in testicular function, sperm quality, reproductive hormone levels, and histological integrity. These potential beneficial protective effects were facilitated by activation of the NRF2/HO‐1 antioxidant pathway, suppression of NF‐κB/NLRP3–associated inflammatory responses, and regulation of apoptosis‐related genes. Collectively, the findings highlight that RA‐CHNPs are a potential nanotherapeutic approach that mitigates CFP‐induced male reproductive toxicity. This encourages adopting nanotechnology‐based antioxidants in reproductive toxicology.

## Author Contributions

**Ahmad Najem Alshammari:** methodology, writing – original draft, Investigation, data curation. **Ayat B Al‐Ghafari:** methodology, writing – original draft, investigation, data curation. **Huda A Al Doghaither:** methodology, writing – original draft, investigation, data curation. **Ahmed M S Hegazy:** validation, formal analysis, writing – review and editing, data curation. **Ekramy M Elmorsy:** conceptualization, funding acquisition, writing – original draft, methodology, writing – review and editing, formal analysis, data curation. **Asmaa Fady Sharif:** conceptualization, writing – original draft, writing – review and editing, methodology, data curation. **Zahraa Khalifa Sobh:** methodology, writing – review and editing, writing – original draft, conceptualization, data curation.

## Conflicts of Interest

The authors declare no conflicts of interest.

## Data Availability

The data that support the findings of this study are available from the corresponding author upon reasonable request.
